# Plasma lipoprotein-associated phospholipase A_2 _activity in Alzheimer's disease, amnestic mild cognitive impairment, and cognitively healthy elderly subjects: a cross-sectional study

**DOI:** 10.1186/alzrt154

**Published:** 2012-12-07

**Authors:** Julie E Davidson, Andrew Lockhart, Leslie Amos, Heide A Stirnadel-Farrant, Vincent Mooser, Marc Sollberger, Axel Regeniter, Andreas U Monsch, Michael C Irizarry

**Affiliations:** 1 Worldwide Epidemiology, GlaxoSmithKline R&D, 1-3 Iron Bridge Road, Stockley Park, UB11 1BT, UK; 2 Memory Clinic, Department of Geriatrics, Basel University Hospital, Schanzenstrasse 55, 4031 Basel, Switzerland; 3 R&D China, GlaxoSmithKline, Addenbrooke's Centre for Clinical Investigation, Addenbrooke's Hospital, Hills Road, Cambridge, CB2 2GG, UK; 4 Genetics, GlaxoSmithKline R&D, 5 Moore Drive, Research Triangle Park, NC 27709, USA; 5 Genetics, GlaxoSmithKline R&D, 709 Swedeland Road, King of Prussia, PA 19406, USA; 6 Laboratory Medicine, Basel University Hospital, Basel, Switzerland; 7 Worldwide Epidemiology, GlaxoSmithKline R&D, 5 Moore Drive, Research Triangle Park, NC 27709, USA

## Abstract

**Introduction:**

Lipoprotein-associated phospholipase A_2 _(Lp-PLA_2_) is a circulating enzyme with pro-inflammatory and oxidative activities associated with cardiovascular disease and ischemic stroke. While high plasma Lp-PLA_2 _activity was reported as a risk factor for dementia in the Rotterdam study, no association between Lp-PLA_2 _mass and dementia or Alzheimer's disease (AD) was detected in the Framingham study. The objectives of the current study were to explore the relationship of plasma Lp-PLA_2 _activity with cognitive diagnoses (AD, amnestic mild cognitive impairment (aMCI), and cognitively healthy subjects), cardiovascular markers, cerebrospinal fluid (CSF) markers of AD, and apolipoprotein E (*APOE*) genotype.

**Methods:**

Subjects with mild AD (*n *= 78) and aMCI (*n *= 59) were recruited from the Memory Clinic, University Hospital, Basel, Switzerland; cognitively healthy subjects (*n *= 66) were recruited from the community. Subjects underwent standardised medical, neurological, neuropsychological, imaging, genetic, blood and CSF evaluation. Differences in Lp-PLA_2 _activity between the cognitive diagnosis groups were tested with ANOVA and in multiple linear regression models with adjustment for covariates. Associations between Lp-PLA_2 _and markers of cardiovascular disease and AD were explored with Spearman's correlation coefficients.

**Results:**

There was no significant difference in plasma Lp-PLA_2 _activity between AD (197.1 (standard deviation, SD 38.4) nmol/min/ml) and controls (195.4 (SD 41.9)). Gender, statin use and low-density lipoprotein cholesterol (LDL) were independently associated with Lp-PLA_2 _activity in multiple regression models. Lp-PLA_2 _activity was correlated with LDL and inversely correlated with high-density lipoprotein (HDL). AD subjects with *APOE*-ε4 had higher Lp-PLA_2 _activity (207.9 (SD 41.2)) than AD subjects lacking *APOE*-ε4 (181.6 (SD 26.0), *P *= 0.003) although this was attenuated by adjustment for LDL (*P *= 0.09). No strong correlations were detected for Lp-PLA_2 _activity and CSF markers of AD.

**Conclusion:**

Plasma Lp-PLA_2 _was not associated with a diagnosis of AD or aMCI in this cross-sectional study. The main clinical correlates of Lp-PLA_2 _activity in AD, aMCI and cognitively healthy subjects were variables associated with lipid metabolism.

## Introduction

Lipoprotein-associated phospholipase A_2 _(Lp-PLA_2_), also known as platelet activating factor acetylhydrolase (PAF-AH), is a circulating enzyme with pro-inflammatory and oxidative activities studied extensively as a marker of cardiovascular risk [1-3]. LpPLA_2 _is measured through assay of either enzyme concentration in the serum (mass) or enzymatic activity [3]. While other cardiovascular risk factors, such as hypertension, hyperlipidemia and diabetes, may increase the risk of developing dementia and Alzheimer's disease (AD) [4], there is limited published epidemiological data regarding the relationship between Lp-PLA_2 _activity and dementia. Individuals aged ≥ 55 years old within the highest quartile of Lp-PLA_2 _activity had an increased risk of developing dementia over a mean follow-up of 5.7 years in the Rotterdam case-cohort study (HR 1.56; CI 1.03 to 2.37); the effect was greater on vascular dementia (HR 2.19, CI 0.80 to 6.03) than for the AD outcome (HR 1.30, CI 0.82 to 2.04) [5]. Lp-PLA_2 _mass (measured as a one standard deviation increase above mean) was not found to be associated with an increased risk of dementia (HR 0.98; CI 0.84 to 1.15) or AD (HR 0.98; CI 0.82 to 1.18) in the Framingham Study, however [6]. Apolipoprotein E (*APOE) *polymorphisms related to AD risk influence Lp-PLA_2 _activity levels [7], yet the null activity polymorphism of the Lp-PLA_2 _gene was not associated with lower risk of AD in a large case-control study in Japan [8].

The principal aim of this study was to examine whether plasma Lp-PLA_2 _activity differed by diagnosis (AD, aMCI, cognitively healthy) in a clinically well characterised group of subjects. Such a finding could be used to support the rationale for the development of Lp-PLA_2 _modifying treatments for use in populations with, or at risk for, dementia. Additional objectives included: (i) exploring the associations between Lp-PLA_2 _and cerebrospinal fluid (CSF) markers of AD, (ii) assessing the association of Lp-PLA_2 _and markers of cardiovascular disease or diabetes in individuals with dementia, and (iii) investigating the relationship between Lp-PLA_2 _and *APOE *genotype.

## Materials and methods

### Subjects

Subjects with AD (by National Institute of Neurological and Communicative Disorders and Stroke - Alzheimer's Disease and Related Disorders Association (NINCDS-ADRDA) [9] criteria, *n *= 78), amnestic MCI (aMCI) (by Petersen criteria, [10]*n *= 59), and cognitively healthy "normal controls" (*n *= 66) were recruited at the Memory Clinic, Department of Geriatrics, University Hospital, Basel, Switzerland, and underwent detailed neuropsychological, clinical, biomarker and imaging assessments at baseline and 12 months post-baseline. All study participants were aged ≥ 50 and had between 7 and 20 years of education. Subjects with AD and aMCI were current clinic patients. Controls were identified from the participants of the Basel Study on the Elderly (BASEL Project) described elsewhere; recruitment was stratified to ensure an even distribution of controls across each decade of age (50 to 59, 60 to 69, 70 to 79 and 80 to 89) and across genders [11]. The study protocol was approved by the University of Basel Institutional Review board and written informed consent was obtained from each patient. The current study is a cross-sectional analysis of data collected at the baseline study visit.

### Laboratory procedures

Plasma aliquots, extracted from baseline blood samples and stored at -80°C, were transferred to Quest Diagnostics Inc. for Lp-PLA_2 _enzymatic activity measurement using an established colorimetric activity method (CAM) [12]. The upper limit of valid measurement for the CAM assay is 300 nmol/min/ml and subjects (*n *= 6) with values greater than or equal to 300 nmol/min/ml were assigned a value of 300 nmol/min/ml. Markers of AD in CSF -- total Tau (T-Tau), phosphorylated Tau, 181P-epitope (P-Tau), and amyloid beta protein 42 (Aß42) -- were collected at the baseline visit and measured by ELISA at the University of Basel using the manufacturer's recommended protocols (Innogenetics NV, Ghent, Belgium).

### Statistical analysis

Differences in mean Lp-PLA_2 _activity between diagnosis groups (AD, aMCI and normal controls) were explored initially using ANOVA. The primary comparison of interest was between AD and normal controls. The study was powered to detect a difference of 20 nmol/min/ml with 90% power and a two-sided alpha of 0.05, assuming a mean (SD) of 144 (36) nmol/min/ml in the normal controls [13].

Potential confounders or modifiers of the relationship between AD and aMCI and Lp-PLA_2 _were explored in a multiple linear regression model using backwards elimination with a retention criterion of *P *< 0.1. Covariates included in the models were statin use (yes/no), age, gender, body mass index (BMI), European Cardiovascular Society (ESC) cardiovascular risk score [14] of > 5%, history of diabetes type 1 or 2, history of heart disease, Hachinksi ischaemia score [15], and white matter changes (Scheltens [16] and Wahlund scores [17]). Statin use, age and gender were forced to remain in the model given demonstrated associations between these factors and dementia or Lp-PLA_2 _activity [18-20].

Lp-PLA_2 _is largely bound to LDL in the circulation, possibly through apolipoprotein B (apoB) 100 [21], and whether or not to adjust analytically for apoB and/or LDL in studies of Lp-PLA_2 _and cardiovascular outcomes is a matter for current scientific debate [3,22]. While it is important to assess whether any observed associations of Lp-PLA_2 _with dementia may simply be proxies for an effect of LDL, controlling for LDL analytically could result in over-correction of the LpPLA_2 _values, obscuring a true association. To address this, the effect of LDL was explored by adjusting the final linear regression model arrived at through backwards elimination for LDL to assess whether this improved the model (assessed by comparison of model R^2^, BIC and AIC). Reporting models both with and without adjustment for LDL is an approach used in the cardiovascular field [3]. Ten subjects with data missing for at least one covariate were dropped from the backwards elimination modelling.

The secondary analyses of the correlates of Lp-PLA_2 _activity were exploratory, and were not adjusted for multiple comparisons. Spearman's correlation coefficients were used to explore the association between Lp-PLA_2 _activity and (i) CSF markers of AD (Aß42, T-Tau and P-Tau), (ii) white matter changes (Scheltens score) and (iii) markers of cardiovascular disease and diabetes (LDL, high-density lipoprotein (HDL), total cholesterol:HDL ratio, homocysteine and haemoglobin A1c (HgbA1C)). Student's *t*-tests were used to test for differences in mean Lp-PLA_2 _by *APOE ε*4 genotype (positive (1 or 2 ε4 alleles) versus negative (0 ε4 alleles)). Multiple linear regression was used to adjust the *APOE ε*4 comparison in the AD group for covariates (statin use, heart disease and LDL; explored in separate models).

All analyses were performed using SAS software, Version 9.1 for Windows. Copyright, SAS Institute Inc. SAS and all other SAS Institute Inc. product or service names are registered trademarks or trademarks of SAS Institute Inc., Cary, NC, USA.

## Results

### Subject demographics

The demographic characteristics of the subjects are shown in Table 1. The groups differed in terms of age (*P *= 0.0005), gender (*P *= 0.02), education (*P *= 0.0005), homocysteine (*P *= 0.002), HDL (*P *= 0.005), Scheltens score [16] (*P *= 0.002), Wahlund score [17] (*P *= 0.04), Hachinski ischemia score [15] (*P *< 0.0001), CSF T-tau (*P *< 0.0001) and CSF P-tau (*P *< 0.0001); values were higher in the AD group than in the normal control group while values in the aMCI group were intermediate. Similarly, differences were detected across the groups for total cholesterol:HDL ratio (*P *= 0.01) and CSF Aβ42 (*P *< 0.0001); values for these measures were lower in the AD group than in the normal control group and, again, values in the aMCI group were intermediate. The mean score on the Mini Mental Status Examination (MMSE) [23] in AD subjects was consistent with mild dementia

**Table 1:** Characteristics of subjects

Characteristic	AD (*n *= 78)	aMCI (*n *= 59)	Normal Control (*n *= 66)	P*
**Demographic**				

Age, y (mean, SD)	75.7 (8.4)	71.3 (8.5)	71.1 (8.3)	0.0005
Female, N (%)	47 (60%)	28 (43%)	24 (36%)	0.017
Education, y (mean, SD)	11.4 (2.8)	12.8 (3.5)	13.1 (2.7)	0.0005

**Clinical**				

Statin Use, N (%)	16 (21%)	17 (28.8%)	11 (17%)	0.26
BMI (mean, SD)	25.4 (3.2)	25.6 (3.9)	24.6 (3.7)	0.33
ESC score (mean, SD)	0.133 (0.110)	0.094 (0.102)	0.103 (0.078)	0.015
Diabetes type 1 or 2, N (%)	6 (7.7%)	1 (1.7%)	3 (4.5%)	0.30
History of heart disease, N (%)	8 (10.3%)	9 (15.3%)	7 (10.6%)	0.68
Hachinski score (mean, SD)	2.6 (1.9)	2.5 (2.6)	0.7 (1.0)	< 0.0001
Scheltens score (mean, SD)	12.2 (9.9)	10.8 (10.8)	6.4 (5.7)	0.0021
Wahlund score (mean, SD)	4.0 (2.7)	3.7 (2.8)	2.8 (1.7)	0.04
Duration of symptoms, y (mean, sd)	2.5 (2.4)	3.1 (3.3)	NA	0.63
MMSE, mean (mean, SD)	23.8 (2.7)	28 (1.7)	29.1 (1.0)	< 0.0001

**Laboratory**				

LpPLA_2_, nmol/min/ml (mean, SD)	197.1 (38.4)	205.5 (43.4)	195.4 (41.9)	0.34
LDL, mmol/L (mean, SD)	2.9 (1.0)	2.9 (0.8)	3.0 (1.2)	0.79
HDL, mmol/L (mean, SD)	2.0 (0.5)	1.8 (0.5)	1.7 (0.5)	0.005
Total cholesterol:HDL ratio	3.0 (0.9)	3.2 (0.9)	3.5 (1.2)	0.012
Homocysteine, Umol/L (mean, sd)	15.1 (5.4)	14.1 (7.9)	12.3 (3.4)	0.002
HbA1c, % (SD)	5.6 (0.4)	5.6 (0.4)	5.5 (0.3)	0.43
CSF markers of AD				
Aβ42, pg/ml (mean, SD)	421.4 (147.6)	568.2 (251.7)	809.7 (260.5)	< 0.0001
CSF T-Tau, pg/ml (mean, SD)	698.5 (317.2)	417.2 (245.6)	308.0 (195.9)	< 0.0001
CSF P-Tau, pg/ml (mean, SD)	101.9 (58.0)	62.9 (27.9)	52.0 (21.6)	< 0.0001

**Genetic**				

*APOE ε4*-positive, N (%)	45 (58%)	30 (50.5%)	4 (6%)	NA**†**

*ANOVA for normally distributed continuous variables, Kruskal-Wallis test for non-normal continuous variables, Fisher's exact test for ordinal variables. † Control selection was not independent of *APOE *status.

### Plasma Lp-PLA_2 _activities

**Figure 1:**
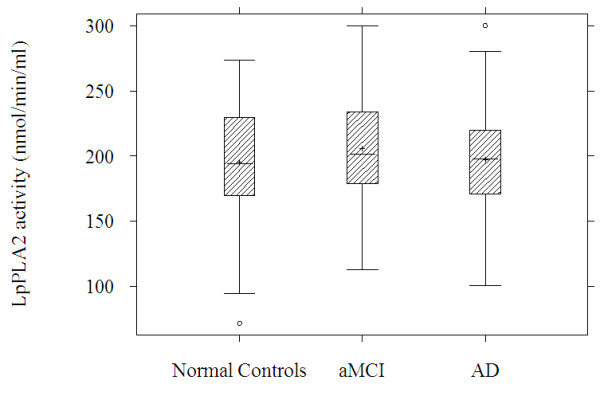
**Lp-PLA_2 _activity by diagnosis group**.

Mean plasma Lp-PLA_2 _activities were generally higher across all groups (195 to 206 nmol/min/ml) compared to those reported previously in the literature (144 to 146 nmol/min/ml) using the CAM assay [13,19] (Table 1 and Figure 1). There was no significant difference in Lp-PLA_2 _activity levels between the control group and the AD group (mean difference 1.7 nmol/min/ml, SD 6.8, *P *= 0.81) or aMCI group (mean difference 10.1 nmol/min/ml, SD 7.4, *P *= 0.17) in the unadjusted comparison.

Results from the backwards elimination multiple linear regression model without LDL and for this model with adjustment for LDL are presented in Table 2. The model with adjustment for LDL had greater explanatory power than the model without LDL (F = 22.39, *P *< 0.0001, R^2 ^= 0.42 versus F = 3.6, *P *= 0.0012, R^2 ^= 0.12, respectively; BIC and AIC both confirmed an improvement in fit). In both models female gender was associated with lower Lp-PLA_2 _activity. Statin use was also associated with lower Lp-PLA_2 _activity levels compared to non-use although this effect was attenuated after adjustment for LDL. In the model without LDL, increased BMI was a significant predictor of Lp-PLA_2 _activity; as with statin use, the effect of BMI was attenuated after adjustment for LDL. LDL was the strongest independent predictor of Lp-PLA_2 _activity in the model with LDL; a 1 mmol/L increase in LDL was associated with a 24.1 nmol/min/ml increase in Lp-PLA_2 _activity. Lp-PLA_2 _activity levels in aMCI trended higher relative to control subjects but the difference only reached statistical significance in the model which included LDL.

**Table 2:** Final linear regression models of Lp-PLA_2 _activity by diagnosis group

	Model Without LDL		Model With LDL	
	**β (SE)†**	***P***	**β (SE)†**	***P***

AD (versus Control)*	7.7 (7.2)	0.29	8.3 (5.7)	0.15
aMCI (versus Control)*	11.3 (7.2)	0.12	**11.6 (5.8)**	**0.05**
Age (1 year increase)*	-0.19 (0.3)	0.58	0.39 (0.3)	0.16
Statin use (yes versus no)*	**-23.9 (7.0)**	**0.001**	-11.2 (5.8)	0.06
Gender (female versus male)*	-10.6 (6.2)	0.09	**-23.8 (4.9)**	**< .0001**
LDL (1 mmol/l increase)	NA	NA	**24.1 (2.5)**	**< .0001**
BMI	**1.8 (0.8)**	**0.03**	0.4 (0.69)	0.55
Diabetes Type 2 (yes versus no)	-23.3 (14.0)	0.10	-2.7 (11.6)	0.81

Final models selected through backwards elimination. Covariates with *P-*value < 0.05 are presented in bold. *Forced to remain in the model. † β estimates correspond to the expected difference in mean Lp-PLA_2 _activity relative to comparator for binary variables or per one unit increase in continuous covariates.

Plasma Lp-PLA_2 _activity did not correlate with CSF biomarkers nor with white matter changes within diagnoses, apart from a marginally significant inverse association with CSF Aβ42 in aMCI (r = -0.29, *P *= 0.03) (Table 3).

**Table 3:** Correlation between plasma Lp-PLA_2 _and (1) CSF biomarkers for AD and (2) Schelten Score for white matter changes

	-ß- amyloid(1-42)	P-Tau (181P epitope)	T-Tau	Schelten Score
	**r (*P*)**	**r (*P*)**	**r (*P*)**	**r (*P*)**
**AD (*n *= 78)**	-0.01 (0.91)	-0.20 (0.08)	-0.10 (0.40)	-0.03 (0.82)
**aMCI (*n *= 59)**	**-0.29 (0.03)**	-0.08 (0.54)	-0.14 (0.28)	0.20 (0.12)
**Normal controls** **(*n *= 28 (CSF tests); *n *= 66 (Scheltens))**	-0.17 (0.39)	0.35 (0.07)	0.27 (0.16)	-0.06 (0.63)

Correlations with *P-*value < 0.05 are presented in bold.

Correlations between plasma Lp-PLA_2 _and plasma markers of cardiovascular disease or diabetes were similar in magnitude and direction across diagnosis groups. Lp-PLA_2 _was correlated with LDL and total cholesterol:HDL ratio (r = 0.51 and 0.54, respectively, *P *< 0.001 in the total pooled sample) and inversely correlated with HDL (r = -0.32, *P *< 0.001). Lp-PLA_2 _was not associated with homocysteine or HgbA1c.

Lp-PLA_2 _levels tended to be higher in individuals who were *APOE ε*4 carriers in each diagnosis group (Table 4) In the AD diagnosis group, mean Lp-PLA_2 _activity was 26.2 nmol/min/l higher in individuals with *APOE ε*4 positive status compared to *APOE ε*4 negative individuals (*P *= 0.003). Adjustment for LDL levels removed this effect, however (*P *= 0.09). LDL levels also tended to be increased in *APOE ε*4 carriers and this difference was greatest in the AD group (*P *= 0.05).

**Table 4:** Lp-PLA_2 _activity and LDL by *APOE ε4 *status

		*APOE *E4+	*APOE *E4-
Normal control (*n *= 66)	Lp-PLA_2_, nmol/min/ml (mean, SD)	206.5 (27.0)	194.7 (42.8)
	LDL, mmol/L (mean, SD)	3.1 (1.3)	3.0 (1.1)
aMCI (*n *= 59)	Lp-PLA_2_, nmol/min/ml (mean, SD)	215.6 (42.1)	195.0 (42.9)
	LDL, mmol/L (mean, SD)	3.1 (0.8)	2.8 (0.8)
AD (*n *= 78)	Lp-PLA_2_, nmol/min/ml (mean, SD)	207.9 (41.2)*	181.6 (26.0)*
	LDL, mmol/L (mean, SD)	3.1 (1.2)*	2.6 (0.6)*

*P < 0.05 (*t*-test; values in bold).

## Discussion

Findings from the Rotterdam Study suggested that Lp-PLA_2_, implicated in cardiovascular disease, could also be a risk factor for dementia [5] whereas analyses from the Framingham Study failed to replicate this association [6]. To our knowledge there have been no previously published studies of plasma Lp-PLA_2 _activity in established aMCI, a potential pre-stage of AD. In the current study, we detected no differences in Lp-PLA_2 _activity levels in cross-sectional comparisons among AD, aMCI and cognitively healthy subjects. Lp-PLA_2 _activity levels were moderately elevated in aMCI relative to control subjects after adjustment for LDL, but this requires further exploration in follow-up studies.

Lp-PLA_2 _activity was found to be associated with carriage of the *APOE ε*4 allele; however, this effect was removed after adjustment for LDL. An association between Lp-PLA_2 _activity and APOE genotype has been reported previously in a single gene study [24]. A recent genome-wide association study found that the G allele of the rs4420638 single-nucleotide polymorphism (SNP) in *APOE *was associated with increased Lp-PLA_2 _activity; the authors suggest that Lp-PLA_2 _activity would be expected to be increased in carriers of *APOE ε*4, given patterns of linkage disequilibrium between rs4420638 and the *APOE ε*2, *ε*3 and *ε*4 genotypes [25]. The correlations observed between Lp-PLA_2 _and LDL and between Lp-PLA_2 _and HDL across the diagnosis groups in our study reflect the findings of previous studies in the general population [19,26,27]. This suggests that mild AD and aMCI are not associated with an alteration in the relationships between these markers.

No strong correlations between Lp-PLA_2 _and CSF biomarkers for AD were observed in our study; the weak inverse association between Lp-PLA_2 _and CSF Aβ42 in the aMCI group (*P *= 0.041) may represent a false positive result from the multiple comparisons performed.

The current study identified the expected associations between AD diagnosis and levels of CSF Aβ42 and tau proteins relative to normal controls [28] and between Lp-PLA_2 _activity and blood lipids, gender and statin use [3]. These results increase confidence around the accuracy of the diagnostic classifications and biochemical measurements. However, the results of the study should be interpreted in the context of potential limitations of the biochemical measurements. First, the mean Lp-PLA_2 _activity observed in the control group (195.4 nmol/min/ml, SD 41.9) was higher than anticipated, based on levels observed in the Framingham Offspring study (144 nmol/min/ml, SD 36) [13] and the Dallas Heart Study (146 nmol/min/ml, SD 40) [19]. These differences may be related to cohort differences, such as the older mean age of the current study population, or assay factors. Secondly, the upper end of the valid range of assay sensitivity was determined by the assay manufacturers to be 300 nmol/min/ml. Six subjects (two with AD and four with aMCI) had Lp-PLA_2 _activity results that exceeded this value and were truncated accordingly. The effect of this truncation on the analyses cannot be quantified but it is likely that the effect would be to bias findings towards the null. Finally, this study evaluated Lp-PLA_2 _activity in plasma. The physiology of the blood-brain barrier and the biochemical complexity of plasma may limit plasma Lp-PLA_2 _activity/mass as a marker of central nervous system physiology [29]. A specific Lp-PLA_2 _activity has recently been reported to be present in human cerebrospinal fluid (CSF) [30] and as this biofluid more closely reflects the composition of the brain extracellular space, may have a higher yield in biomarker evaluation [31].

## Conclusions

The principal correlates of Lp-PLA_2 _activity in analyses of our small cross-sectional study were variables involved in lipid metabolism (LDL, HDL, total cholesterol:HDL ratio and statin use) and variables influencing lipid metabolism (*APOEε*4 and gender). Although there were suggested associations between aMCI and elevated Lp-PLA_2 _levels, and between CSF Aβ42 and Lp-PLA_2 _activity in the aMCI group, the results must be interpreted with caution until replicated in further studies given the small sample size and multiple comparisons associated with the current study.

## Abbreviations

AD: Alzheimer's disease; aMCI: Amnestic mild cognitive impairment; *APOE*: apolipoprotein E; Aβ42: amyloid beta-42; BMI: Body mass index; CAM: Colorimetric activity method; CSF: cerebrospinal fluid; HDL: High-density lipoprotein; HgbA1C: haemoglobin A1c; LDL: Low-density lipoprotein; Lp-PLA_2_: Lipoprotein-associated phospholipase A_2_; MMSE: Mini Mental Status Examination; NINCDS-ADRDA: National Institute of Neurological and Communicative Disorders and Stroke - Alzheimer's Disease and Related Disorders Association; PAF-AH: Platelet activating factor acetylhydrolase; P-Tau: Phosphorylated tau; SD: Standard deviation; SNP: Single-nucleotide polymorphism; T-Tau: Total tau.

## Competing interests

JED, LA, AL, HAS-F, VM and MCI were employees of GlaxoSmithKline at the time of writing. JED, AL, HAS-F, VM and MCI were also stockholders of GlaxoSmithKline plc.

## Authors' contributions

JED designed the current study analysis, performed the data analyses and drafted the manuscript. AL, LA, HS-F, VM and MCI contributed to the study design, interpretation of results and manuscript drafting. MS, AR and AUM collected data, and contributed to interpretation of results and manuscript drafting. All authors read and approved the final manuscript.
